# Preparation, Characterization, and Pharmacological Activity of *Cymbopogon winterianus* Jowitt ex Bor (Poaceae) Leaf Essential Oil of *β*-Cyclodextrin Inclusion Complexes

**DOI:** 10.1155/2015/502454

**Published:** 2015-07-13

**Authors:** Priscila L. Santos, Adriano A. S. Araújo, Jullyana S. S. Quintans, Makson G. B. Oliveira, Renan G. Brito, Mairim R. Serafini, Paula P. Menezes, Marcio R. V. Santos, Pericles B. Alves, Waldecy de Lucca Júnior, Arie F. Blank, Viviana La Rocca, Reinaldo N. Almeida, Lucindo J. Quintans-Júnior

**Affiliations:** ^1^Postgraduate Program in Health Science, Federal University of Sergipe, 49060-100 Aracaju, SE, Brazil; ^2^Department of Chemistry, Federal University of Sergipe, 49100-000 São Cristóvão, SE, Brazil; ^3^Department of Morphology, Federal University of Sergipe, 49100-000 São Cristóvão, SE, Brazil; ^4^Department of Agronomy, Federal University of Sergipe, 49100-000 São Cristóvão, SE, Brazil; ^5^Postgraduate Program in Natural Products and Bioactive Synthetics (PgPNSB), Federal University of Paraíba, 58051-900 João Pessoa, PB, Brazil

## Abstract

This study aimed to evaluate the orofacial antinociceptive effect of the *Cymbopogon winterianus* essential oil (LEO) complexed in *β*-cyclodextrin (LEO-CD) and to assess the possible involvement of the central nervous system (CNS). The LEO was extracted, chromatographed, and complexed in *β*-cyclodextrin. The complex was characterized by differential scanning calorimetry (DSC) and thermogravimetry derivative (TG/DTG). Male Swiss mice (2-3 months) were treated with LEO-CD (50–200 mg/kg, p.o.), vehicle (distilled water, p.o.), or standard drug (i.p.) and subjected to the orofacial nociception formalin-, capsaicin-, and glutamate-induced. After the formalin test, the animals were perfused and the brains subjected to immunofluorescence for Fos. The rota-rod test (7 rpm/min) was carried out. Geraniol (37.57%) was the main compound of LEO. DSC and TG/DTG proved the complexation. The orofacial nociceptive behavior was significantly (*p* < 0.05) reduced. The number of Fos-positive cells was significantly changed in the dorsal raphe nucleus (*p* < 0.01), locus coeruleus (*p* < 0.001), trigeminal nucleus (*p* < 0.05), and trigeminal thalamic tract (*p* < 0.05). LEO-CD did not cause changes in motor coordination in the rota-rod test. Thus, our results suggested that LEO-CD has an orofacial antinociceptive profile, probably mediated by the activation of the CNS without changing the motor coordination.

## 1. Introduction

Pain is defined by the International Association for the Study of Pain (IASP) as an unpleasant sensory and emotional experience associated with actual or potential tissue damage or described in terms of such damage [1]. The orofacial pain, which has a central origin, has emerged as a worldwide health problem. It is estimated that over 22% of Americans, over 18 years, experience pain in the orofacial region. This prevalence is repeated in countries such as England and Germany [2]. Various medicaments have been proposed in an attempt to alleviate orofacial pain; however, many patients do not respond adequately to this type of treatment, demonstrating the need for further studies in order to obtain effective drugs regarding this pathology [3].

Natural products, such as medicinal plants, have been a source of new therapeutic proposals in an attempt to obtain new drugs or precursors with a specific formulation [4]. Studies have demonstrated the effect of natural products on several pathologies and symptoms. In this context are the terpenes and monoterpenes inserted for the pain treatment [5–8].

The use of cyclodextrins (CDs), which are cyclic oligosaccharides, has been described in oral pharmaceutical formulations by means of the formation of inclusion complexes. Several advantages for the drugs are established such as solubility, dissolution rate, stability, bioavailability enhancement, modification of the drug release site and/or time profile and reduction or prevention of gastrointestinal side effects as well as unpleasant smell or taste, or even the conversion of oil and liquid drugs into microcrystalline or amorphous powders [9–11]. According to Tiwari et al. [12], about 30 medicines contain in their formulas the CDs available in the pharmaceutical market, such as the tiaprofenic acid, nimesulide, omeprazole, and cephalosporin normally used in the clinical setting [13]. In order to solubilize nonpolar molecules, or complex mixtures such as essential oils, the formation of inclusion complexes with CDs has been extensively used [10, 14, 15]. Following this approach, our group has recently studied the complexation of natural products, mainly terpenes or essential oils with CDs, seeking to improve the chemical and pharmacological properties of these compounds [15–18].

*Cymbopogon winterianus* Jowitt ex Bor is a tropical plant with various pharmacological applications including its main compounds (citronellal, citronellol, and geraniol), such as cardiovascular [19], anti-inflammatory, antinociceptive [6, 20], and central nervous system (CNS) disorders [21, 22]. However, the analgesic effect of this oil on orofacial pain models still has not been studied. The CNS areas activated after* C. winterianus* leaf essential oil (LEO) administration and its antinociceptive profile are little studied although it continues to be used in folk medicine to treat pain and inflammation, which indicates its high pharmacological and biotechnological potential. Additionally, its short half-life and poor solubility in water have limited the application in medicine.

Hence, we aimed to evaluate the orofacial antinociceptive profile as well as the CNS involvement of LEO complexed in *β*-cyclodextrin (LEO-CD) in rodents, being the first study in the literature that reports the complexation of LEO in *β*-CD, its assessment in CNS areas using protein Fos protein as tool, and evaluation of its effect on orofacial pain animal models.

## 2. Materials and Methods

### 2.1. Chemicals

Chemicals are Alexa Fluor 488 (Life Technologies, Carlsbad, California, USA); c-fOS polyclonal antibody (Santa Cruz Biotechnology, Dallas, Texas, USA); capsaicin (Sigma, USA); formalin (UFS, Brazil); gelatin (UFS, Brazil); glutamate (Sigma, USA); ketamine (Cristália, Brazil); morphine (Merck, Brazil); solution of sodium chloride (UFS, Brazil); Bovine Serum Albumin (BSA) (Santa Cruz, USA); sucrose solution (UFS, Brazil); phosphate buffered saline (UFS, Brazil); and xylazine (Cristália, Brazil).

### 2.2. Obtaining of the Essential Oil

The LEO was obtained by collecting the leaves of the same plant (Arie Blank, ASE9389) at the Federal University of Sergipe, Brazil, Station of Research of the “Rural Campus of the UFS” (latitude 11°00′S and longitude 37°12′W). The leaves with essential oil (LEO) were obtained through hydrodistillation in a Clevenger apparatus, in which the leaves of* C. winterianus* were deposited and dried in an oven with circulation and renewal of air (MA-037/18 model) at 40°C to achieve complete dehydration.

### 2.3. Gas Chromatography (GC)

GC analyses were performed using the gas chromatography equipment coupled to a mass spectrometer with flame ionization detector (GC-MS/FID) (GC-2010 Plus, GCMS-QP2010 Ultra, Shimadzu Corporation, Kyoto, Japan) equipped with an autosampler AOC-20i (Shimadzu). The separations were carried out using a Restek Rtx-5MS fused silica capillary column (5% polysiloxane-diphenyl-95% dimethyl) of 30 m × 0.25 mm internal diameter (di) and 0.25 mm in film thickness at a constant flow of helium (99.999%) with a rate of 1.2 mL min^−1^. The oven temperature program used was from 50°C (isothermal for 1.5 min) with an increase of 4°C/min, up to 200°C, and then 10°C/min to 250°C ending with 5 min isotherm at 250°C.

The FID and MS data were simultaneously acquired using a system of separation detector. The split ratio was 4 : 1 flow (MS : FID). A tube restrictor 0.62 m × 0.15 mm di (capillary column) was used to connect the splitter to the MS detector. A tube restrictor 0.74 m × 0.22 mm i.d. was used to connect the splitter to the FID. The injector temperature was 250°C and the ion source temperature was 200°C. A mass spectrum was generated at 70 eV with a scan speed of 0.3 sec scan being detected in the range of 40–350. The FID fragment temperature was adjusted to 250°C and the supply of gas to the FID was synthetic air, hydrogen, and helium flow rates of 30, 300, and 30 mL·min^−1^, respectively. The quantification of each constituent was estimated by normalizing the peak of the FID generated (%) area. The concentrations of the compounds were calculated from the GC peak areas and were arranged in order of elution from the GC.

The identification of the components was performed based on the comparison of the retention index described in the literature [23]. In order to calculate the retention index (RI), the Van Den Dool and Kratz [24] ratio equation for a homologous series of n-alkanes (nC9–nC18) was used. Three libraries, WILEY8, NIST107, and NIST21 equipment which allow the comparison of the spectral data with those contained in the libraries, using a similarity index of 80%, were also used.

### 2.4. Preparation of the Inclusion Complex

The complex was prepared according to the procedure described by Pinto et al. [25], which consisted of the following steps: (1) physical mixture (PM): LEO (taking into account the weight of the major component LEO, geraniol, 154.24 g/mol) and *β*-CD (1135 g/mol) were mixed (1 : 1 mole ratio) mechanically at ambient conditions, (2) kneading (KN): the LEO and *β*-CD were mixed (1 : 1 mole ratio) with then adding 2.0 mL of water and homogenizing the preparation in mortar and pestle, and (3) coevaporation (CE): LEO and *β*-CD were mixed (1 : 1 mole ratio) in 60 mL of water under constant stirring (36 h) in 400 rpm until equilibrium. Hence, the samples were dried in a glass desiccator and after that were removed and stored in amber vials.

### 2.5. Characterization of Complex

The samples were subjected to differential and scanning calorimetry (DSC), thermogravimetry derivative (TG/DTG). The DSC curves were obtained on DSC-50 cell (Shimadzu) using aluminum sample holder with 2 mg of sample in an atmosphere of nitrogen (50 mL/min^−1^) and heating rate of 10°C min^−1^ in the temperature range from 25 to 500°C. The DSC cell was checked with indium (RM 156.6°C, ΔHmel = 28.54 Jg-1) and zinc (PF 419.6°C). The TG/DTG curves were obtained in thermobalance model TGA 50 (Shimadzu) using a temperature range of 25–900°C in a platinum sample holder with ~3 mg of sample under dynamic nitrogen atmosphere (50 mL/min^−1^) and heating rate of 10°C/min^−1^. The thermobalance was verified with CaC_2_O_4_·H_2_O.

### 2.6. Scanning Electron Microscopy (SEM)

The dried products were mounted and visualized with a JEOL Model JSM-7410-F scanning electron microscope, at an accelerated voltage of 1 kV.

### 2.7. Animals

Adult (3-month-old) male albino Swiss mice (28–32 g) were randomly housed (*n* = 6/per group) in appropriate cages at 21 ± 2°C with a 12 hr light: dark cycle (light from 06:00 to 18:00), with free access to food (Purina, Brazil) and tap water. All experiments were carried out between 09:00 am and 02:00 pm in a quiet room. Experimental protocols were approved by the Animal Care and Use Committee at the Federal University of Sergipe (CEPA/UFS number 42/12). The ethical principles established by the Brazilian Society for Laboratory Animal Science (SBCAL) and by the National Institutes of Health (NIH) were respected. All tests were carried out by the same blinded visual observer and all efforts were made to minimize the number of animals used as well as minimize any discomfort. Vehicle and experimental drug were performed orally by gavage.

### 2.8. Formalin-, Capsaicin-, and Glutamate-Induced Orofacial Nociception

These tests consisted of the subcutaneous injection of formalin (20 *μ*L; 2%) [26], capsaicin (20 *μ*L, 2.5 *μ*g), or glutamate (40 *μ*L, 25 *μ*M) [27] on the right upper lip. Initially, each animal was placed in a mirrored box (30 × 30 × 30 cm) for 30 min to minimize stress. Then, the injection of formalin, capsaicin, or glutamate was performed and, immediately after that, the animals returned to the box for the observation period, divided into 2 blocks of 0–5 min and 15–30 min, 1 block of 42 min or 1 block of 15 min for the formalin, capsaicin, and glutamate tests, respectively. The nociceptive intensity was determined by measuring the time (s) that the animal spent face-rubbing in the injected area with its fore or hind paws. In order to assess the effect of the test drug, mice were pretreated with LEO-CD (50, 100, and 200 mg/kg, p.o.), distilled water (p.o.), or morphine (MOR, 5 mg/kg, i.p.), 1 h before the local injection of the agents.

### 2.9. Assessment of the Motor Activity

In order to evaluate the possible nonspecific muscle-relaxant or sedative effects of LEO-CD in the doses used, mice were subjected to rota-rod apparatus (AVS, Brazil) [28], adapted by Quintans-Júnior et al. [27]. The animals were selected 24 h previously, eliminating those mice which did not remain on the bar for two consecutive periods of 180 s. Animals were treated with LEO-CD (50, 100, and 200 mg/kg, p.o.), vehicle (distilled water, p.o.), or diazepam (DZP, 1.5 mg/kg, i.p.) and observed at times of 30, 60, 120, and 180 min after treatment in a rotating frequency of 7 rpm and for 180-second bar. The results are expressed as the average time(s) the animals remained on the rota-rod in each group.

### 2.10. Immunofluorescence for Fos

Based on studies conducted by Barr [29], immediately after the formalin-induced orofacial nociception test, the animals were perfused and had their brains removed and cryoprotected for immunofluorescence processing to Fos protein as described by Brito et al. [30].

Frozen serial transverse sections (20 *μ*m) of all brains were collected on gelatinized glass slides. The tissue sections were stored at −80°C until use. The sections were washed with phosphate buffer (0.01 M) saline isotonic (PBS) 5 times for 5 min and incubated with 0.01 M glycine in PBS for 10 min. Nonspecific protein binding was blocked by incubation of the sections for 30 min in a solution containing 2% BSA. Then, the sections were incubated overnight with rabbit anti-Fos as primary antibodies (k-25; 1 : 2000). Afterwards, the sections were incubated for two hours with donkey anti-rabbit Alexa Fluor 488 as secondary antibodies (1 : 2000). The cover slip was mounted with glycerol solution. As an immunofluorescence control for nonspecific labeling, sections were incubated without primary antibody. After each stage, slides were washed with PBS 5 times for 5 min.

### 2.11. Acquisition and Analyses of Images

Pictures from Fos-positive areas were acquired for each animal with an Olympus IX2-ICB (Tokyo, Japan). The brain regions were classified according to Paxinos and Franklin Atlas [31]. Neurons were counted by the free software Image J (National Institute of Health) using a plug-in that uses the same level of label intensity to select and count the Fos-positive cells [30].

### 2.12. Statistical Analysis

Results were expressed as mean ± S.E.M. Differences between groups were analyzed using one-way analysis of variance (ANOVA) followed by Tukey's test. In all cases, differences were considered significant if *p* < 0.05. The statistical analyses were assessed using the GraphPad Prism 5.0 software (GraphPad Prism Software Inc., San Diego, CA, USA).

## 3. Results

The qualitative analysis of the LEO sample through GC-MS allowed observing the presence of 20 components. The major constituents of the essential oil were geraniol (37.57%), citronellal (27.09%), geranial (9.63%), citronellol (9.53%), and neral (7.32%) (Table 1).

**Figure 1:**
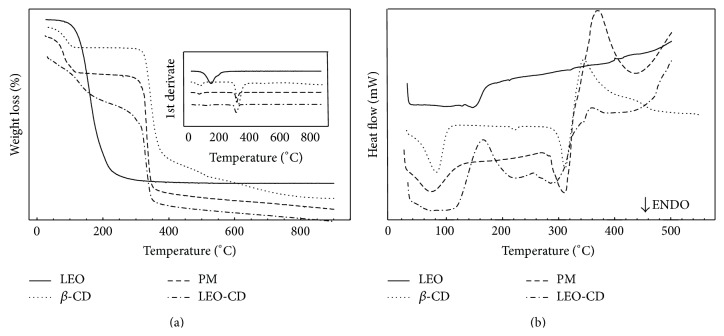
Curve of mass loss (%) (a) and of the heat flow (b) versus temperature (°C) of essential oil of* C. winterianus* Jowitt ex Bor (LEO) and *β*-cyclodextrin (*β*-CD) alone and in physical mixture (PM) process and coevaporation (CE).

The DSC curve of LEO exhibited an endothermic event at 146°C followed by decomposition with loss of mass (Δ*m*) of 93.3%, while the DSC curve of *β*-CD showed three endothermic events (27–120°C: Δ*m* = 12.51%, 212–232°C: Δ*m* = 277–345°C: Δ*m* = 72.88%), followed by an exothermic event (Δ*m* = 10.76%). The coevaporation DSC curve made three endothermic events followed by decomposition. In curves, TG/DTG results are expressed in these four stages of mass loss, in a similar manner to PM. However, in the temperature range of 120–270°C, the TG/DTG curve of CE showed a mass loss of 18.02%, while the PM had a percentage mass loss of only 1.99%. The DSC curve of PM showed two endothermic events (27–152°C and 269–326°C) (Figure 1). The TG/DTG curve confirms this result showing four stages of mass loss of MF (Table 2), which indicates the complexation of LEO in *β*-CD (LEO-CD).

**Figure 2:**
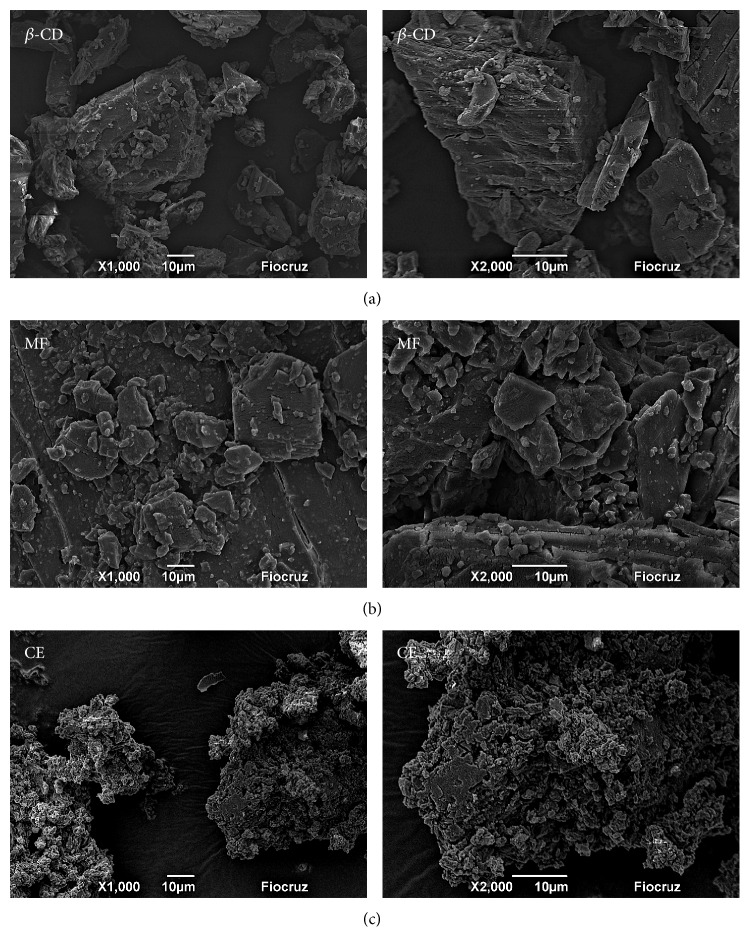
SEM micrographs of cross sections of (a) *β*-CD, (b) physical mixture (PM), and (c) slurry complex (SC). 1000x and 2000x.

SEM micrographs show the different magnifications of the *β*-CD, physical mixture (PM), and slurry complex (SC). By using the SEM, in Figure 2(a), one can see the different sizes of rectangular-shaped and small particles that adhered to the surfaces of the crystals.  Figure 2(b) shows an aspect similar to Figure 2(a), which demonstrates the low capacity of molecular inclusion. However, Figure 2(c) allows observing the amorphous structure. The original morphology of the raw materials disappeared and this property is attributed to the formation of inclusion complexes.

**Figure 3:**
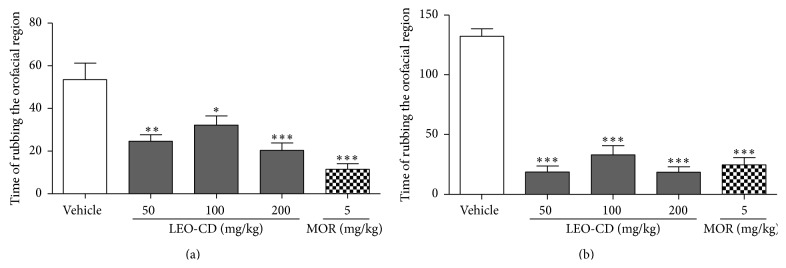
Effect of leaf essential oil of* C. winterianus* Jowitt ex Bor complexed in *β*-cyclodextrin (LEO-CD) on the formalin-induced orofacial nociception. Vehicle (control), LEO-CD (50, 100, and 200 mg/kg), or morphine (MOR 5 mg/kg) was administered orally 60 min before the formalin injection. (a) First phase (0–5 min) and (b) second phase (15–30 min). Values expressed as mean ± S.E.M. (*n* = 6 per group). ^*∗*^ *p* < 0.05, ^*∗∗*^ *p* < 0.01, or ^*∗∗∗*^ *p* < 0.001 when compared with control (ANOVA, one-way, followed by Tukey test).

Administration of LEO-CD produced a reduction in the face-rubbing behavior induced by formalin (Figure 3). All the doses of LEO-CD tested significantly (*p* < 0.05) reduced nociceptive behavior in the first and in the second phase when compared with control (vehicle).

**Figure 4:**
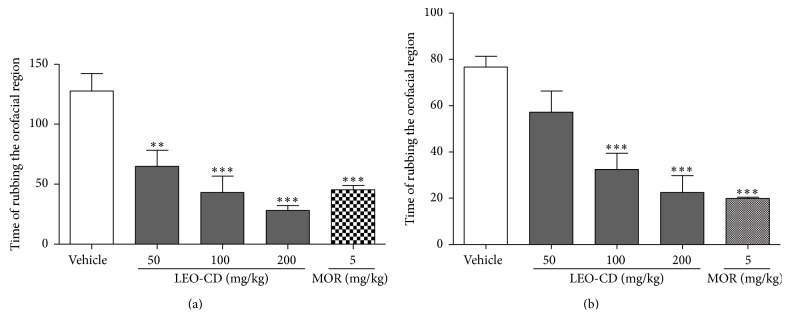
Effect of leaf essential oil of* C. winterianus* Jowitt ex Bor complexed in *β*-cyclodextrin (LEO-CD) on the capsaicin-induced (a) or glutamate-induced (b) orofacial nociception. Vehicle (control), LEO-CD (50, 100, and 200 mg/kg), or morphine (MOR 5 mg/kg) was administered orally 60 min before the injection of capsaicin or glutamate. Values expressed as mean ± S.E.M. (*n* = 6 per group). ^*∗∗*^ *p* < 0.01 or ^*∗∗∗*^ *p* < 0.001 when compared with control (ANOVA, one-way, followed by Tukey test).

In the orofacial nociception induced by capsaicin, LEO-CD, in all doses, significantly decreased (*p* < 0.01) the face-rubbing time (nociceptive behavior) when compared with the control group (Figure 4(a)). Similar effects were observed in the glutamate-induced nociception test, where LEO-CD reduced significantly (*p* < 0.001), at doses of 100 and 200 mg/kg, the nociceptive behavior when compared with the vehicle group (control) (Figure 4(b)). The reference drug, morphine, significantly produced (*p* < 0.001) face-rubbing time in both protocols.

In the rota-rod test, LEO-CD-treated mice, in all doses, did not show any significant motor performance alteration (data not shown). The CNS depressant drug, diazepam (1.5 mg/kg, i.p., standard drug), reduced the time of treated animals on the rota-rod apparatus, as it might be expected.

**Figure 5:**
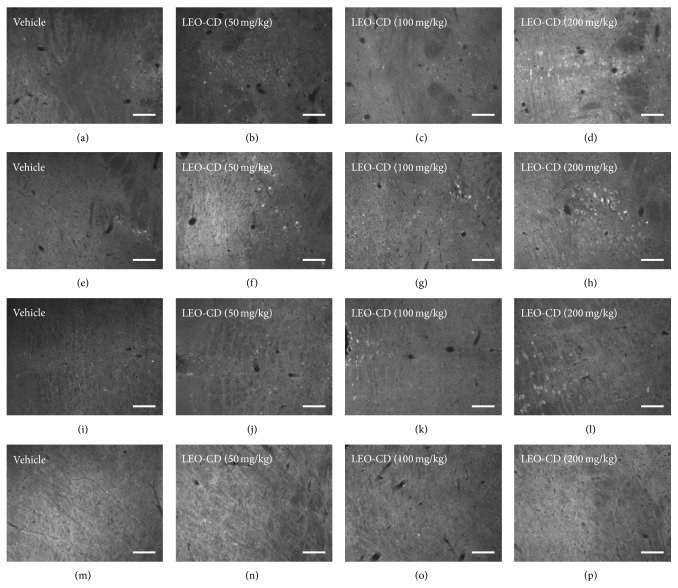
Immunofluorescence for Fos protein in neurons of brain of mice. Dorsal raphe nucleus (a, b, c, and d), locus coeruleus (e, f, g, and h), trigeminothalamic tract (i, j, k, and l), and trigeminal nucleus (m, n, o, p) ninety minutes after treatment and 30 minutes after algesic induction with formalin. 20 *μ*m.

**Figure 6:**
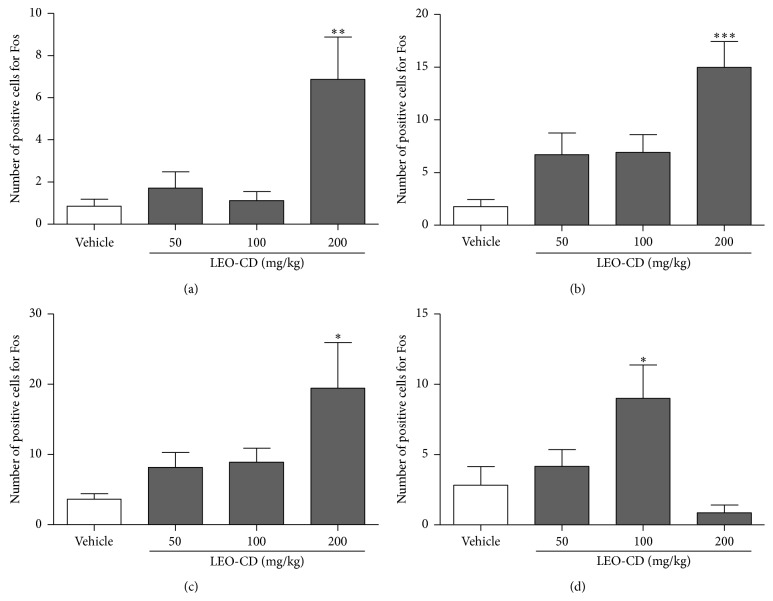
Number of positive cells for Fos in the dorsal raphe nucleus (a), locus coeruleus (b), trigeminothalamic tract (c), and spinal trigeminal nucleus (d). Vehicle (distilled water, p.o.) or LEO-CD (50, 100, and 200 mg/kg, p.o.) was administered ninety minutes before the perfusion. Values expressed as mean ± S.E.M. (*n* = 6 per group). ^*∗*^ *p* < 0.05, ^*∗∗*^ *p* < 0.01, and ^*∗∗∗*^ *p* < 0.001 when compared with control (ANOVA one-way followed by Tukey test).

Pretreatment with LEO-CD produced significant neuronal activation (*p* < 0.05) by immunohistochemistry for c-fos protein in the dorsal raphe (DR) nucleus, locus coeruleus (LC), trigeminal nucleus (TN), and trigeminothalamic tract (TTT) when compared to the control group (Figures 5 and 6).

## 4. Discussion

The genus* Cymbopogon*, belonging to the family Poaceae, is rich in essential oil of aromatic character [21].* C. winterianus* Jowitt ex Bor, popularly known as “citronella grass,” is one of the main Brazilian highlights and it is a medicinal plant species widely distributed in northeastern Brazil [32]. Some pharmacological properties attributed to LEO have been described by our group, such as cardiovascular, analgesic, and anti-inflammatory [19, 20, 33] properties. However, low water solubility, short half-life, and low absorption by oral administrations have limited pharmacological uses of essential oils or main compounds [10, 15]. Thus, we assessed the effect of LEO complexed in *β*-CD on orofacial nociception protocols and its action on the CNS by immunofluorescence for Fos protein.

Our study demonstrated that LEO possesses a chromatographic profile similar to that presented by Leite et al. [20, 33] and de Menezes et al. [19], in which the major components of LEO are geraniol, citronellal, geranial, citronellol, and neral. According to Stotz and collaborators [34] that showed that the geraniol, geranial, and neral act in vanilloid receptor 1, indicating the possible inhibitory effect of these compounds on the painful behavior. In addition, the effect of citronellol [30] and citronellal [27] on orofacial pain models has also been described with pharmacological mechanisms which may be considered innovators to monoterpenes, such as sodium channel blockers and involving the descending pain-inhibitory mechanisms. Therefore, we proceeded with the complexation of LEO with cyclodextrin and performed the characterization of these inclusion complexes to the best of our knowledge for the first time in literature. The endodermal event observed on the DSC curve of LEO is typical of the volatilization of oil, which can be seen by the curves TG/DTG, where the mass loss of the oil is observed.

The DSC curve of *β*-CD resulted in three endothermic events. The first is referring to the release of water molecules in their molecular structure, the second is a characteristic crystalline phase transition where there is no mass change, being a physical phenomenon, and the third is related to the melting followed by decomposition of the material. Thereafter, it began to decompose exothermically with the formation of elemental carbon, which is the fourth stage of mass loss. The DSC curve of the PM showed two endothermic events. The range of 27–152°C comprises the sum of the phase of water release from the *β*-CD molecule and volatilization of LEO, and the temperature range of 269–326°C is an observed process of degradation.

These results are a clear evidence of the formation of inclusion complexes, since the oil encapsulated in *β*-CD, by the CE method, can not volatilize at temperatures below 120°C. As it needs a higher temperature for this process to occur, it is possible to suggest the presence of oil in the cavity of *β*-CD. However, in the PM, the volatilization of LEO occurred in the first stage of mass loss, which has a 21.06% removal of water molecules as well as LEO possibly adsorbed on the surface of *β*-CD [15, 35]. Thus, we obtained a novel complex formed by LEO with *β*-CD. In addition, the dose tested in our protocols is substantially smaller than the smallest dose of LEO described in the literature by Leite et al. [20], because we used the 1 : 1 molar ratio of majority compound (geraniol: 154.24 g/mol) and *β*-CD (1135 g/mol), so we are using nominally around 10% of the dose used by Leite et al. [20]. Therefore, this complex seems to present better analgesic effect using lower concentrations of active ingredient, with these benefits being already described for cyclodextrins [36, 37].

In addition, to evaluate the possible orofacial antinociceptive profile of LEO-CD, some protocols were carried out. The first protocol was the formalin-induced nociception test. This test is the most appropriate test for central review, being divided into two phases [26]. The first phase refers to a direct stimulation of nociceptors by the release of substance P (SP), whereas the second leads to an inflammatory process with subsequent central sensitization [38, 39]. When injected in the orofacial area, formalin activates the trigeminal nociceptive fibers [40].

The antinociceptive effect of LEO-CD observed in the first phase may be related to the inhibition of the SP release. Our data corroborate with the results of Leite et al. [20], which tested the antinociceptive effect of LEO in formalin test when injected in the paw. According to Su et al. [41], geraniol and citronellol act in the suppression of NO production by the modulation of iNOS enzyme and reduced expression of cyclooxygenase 2 (COX-2), suggesting these possible actions to LEO-CD in the second phase, which corroborate with the anti-inflammatory activity already described for the LEO [20].

Capsaicin acts on sensory fibers responsible for pain transmission and activates the transient receptor potential vanilloid subtype 1 (TRPV1) [42], an ion channel sensitive to heat, pH acid [43], lipid mediators [44, 45], and several natural products [46–48]. So, the decreased friction of the orofacial region in mice following administration of LEO-CD in the capsaicin-induced orofacial nociception test may be related to the TRPV1 agonist effect already described for citronellol [49], geraniol [34, 49], geranial, and neral [34].

Previous studies using ketamine, an antagonist of NMDA receptor, on the temporomandibular joint (TMJ), showed a significant decrease in glutamate-induced pain. As this neurotransmitter is able to activate primary afferent nociceptors after its release from inflamed tissues [50], the LEO-CD may be acting as an inhibitor of the glutamatergic system, since the frictional behavior was reduced when glutamate was injected, corroborating the results described by Silva et al. [22].

Studies have suggested that the CNS depression and the nonspecific muscle relaxation effects can reduce the response of motor coordination and might invalidate the behavioral tests [40, 51]. However, previous studies with LEO did not show any interference on the motor coordination of the animals in the rota-rod test [33], corroborating with our results with LEO complexed in CDs. Therefore, the action of LEO-CD on orofacial nociception tests, observed in this study, is not entirely due to impairment as CNS depression or muscle relaxation.

In order to investigate which brain areas are activated after LEO-CD administration, we performed, in an unprecedented manner for this active compound, the immunofluorescence protocol for Fos protein [52]. This protein can be used as a target due to its release after activation of neurons, which allows its use as a marker of neuronal activity [53]. The study of the Fos expression is indicated when the research aims to investigate the possible mechanisms involved in the perception and response to pain originating from the CNS [30, 54]. After the evaluation of the Fos expression evoked by LEO-CD administration, we observed the activation of the trigeminal nucleus, trigeminothalamic tract, locus coeruleus, and dorsal raphe nucleus.

The orofacial nociception test induced by formalin, which preceded the removal of the tissue for subsequent submission to immunofluorescence for Fos protein, occurred due to the application of a painful stimulus in the jaw area. This area is innervated by the trigeminal nerve, which connects with the trigeminal nucleus (TN) [40]. In this nucleus, there was the expression of the Fos protein in the intermediate dose, but there was no such an expression in the higher dose. Information among different CNS areas is generally transmitted by tracts, like the trigeminothalamic tract (TTT). The TTT is also known as trigeminal lemniscuses, sending afferents from TN to the thalamus, mediated by the glutamatergic system [55, 56], and also sending projections to the primary sensory cortex in order to follow the inhibitory pathway of pain, resulting in analgesia [57, 58]. The expression of Fos protein in these areas suggests that LEO-CD has been acting through these pathways.

Studies with medicinal plants have showed the effect of these plants in their intermediate dose, but not in their higher dose, as occurred in our study. This effect has been described for medicinal plants as hormesis effect. Our suggestion is that the saturation of receptors by bioactive compounds present in the high dose of LEO-CD can be inducing the mechanism of downregulation [59].

The locus coeruleus (LC) is involved in the processing and inhibition of pain and has been used in many classic studies of acute pain modulation [60]. A recent study showed the importance of glutamate in the descending inhibition in rats [61]. According to Nygren and Olson [58], LC would receive information from somatosensorial cortex and inhibit the pain by descendent pathway. Therefore, data obtained with the test of nociception induced by glutamate and previous study by our research group with the LEO [21] suggest the involvement of the glutamatergic system in the LEO-CD-action.

A previous study using an antagonist of the serotonin (5-HT) receptor demonstrated the reduction of nociceptive sensitivity after the formalin-induced orofacial nociception tests, on the inflammatory phase. This suggests the serotonergic pathway as one of the likely routes of nociceptive stimuli involved in this nociceptive protocol [62]. This study suggests that LEO-CD would activate also the serotoninergic pathway by expression of Fos protein in the dorsal raphe (DR) nucleus [63], which activate the pain descending pathway [64].

Through the immunofluorescence data, it was possible to show that the LEO-CD was able to activate the trigeminal nucleus, trigeminal thalamic tract, and locus coeruleus in the CNS, possibly by the glutamatergic pathway, confirming the data observed in the test of glutamate-induced nociception. Silva et al. [22] demonstrated previously the activity of the LEO on the glutamatergic system. Our study also demonstrated the action of LEO-CD in the dorsal raphe nucleus, which has great influence of the serotonergic routes [63]. This suggests the action of the LEO-CD in the descending pain pathway, particularly in specific areas responsible for the transmission of impulses related to orofacial pain, such as trigeminal pathway.

In summary, the use of LEO complexed in CD reduces considerably the dose of LEO contained in the complex when compared to the doses commonly used for the pure LEO described in the literature. Additionally, we observed the analgesic activity of LEO-CD on orofacial nociception and activation of CNS areas, specifically regions, directly or indirectly, involved in the descendent pain pathway, different from most drugs available in the market for the treatment of the pain nowadays. Thus, it can be concluded that the LEO-CD can be a biotechnological option to be implanted, in the future, for pain management, including orofacial pain, confirming the importance of the natural products in the world health as a potential source of new drug substances.

## Figures and Tables

**Table 1:** Chromatographic profile of leaf essential oil of *C. winterianus* Jowitt ex Bor (LEO) by GC-MS.

Peak	TR (min)	Compounds	(%) GC-MS	(%) GC/FID	IRR exp.^*∗*^	IRR lit.^*∗∗*^
1	10.505	6-Methyl-5-hepten-2-one	0.22	0.22	988	981
2	12.060	Limonene	1.36	2.03	1031	1024
3	12.980	Bergamal	0.26	0.33	1056	1051
4	13.675	NI	0.31	0.30	1074	NI
5	14.705	Linalool	0.58	0.73	1102	1095
6	15.815	NI	0.17	0.24	1131	NI
7	16.520	Isopulegol	0.42	0.43	1150	1145
8	16.795	Citronellal	27.09	28.55	1157	1148
9	17.180	Z-Isocitral	0.31	0.22	1167	1160
10	17.875	E-Isocitral	0.31	0.34	1185	1177
11	18.710	n-Decanal	0.34	0.34	1208	1201
12	19.575	Citronellol	9.53	10.14	1231	1223
13	20.095	Neral	7.32	6.94	1246	1235
14	20.615	Geraniol	37.57	35.43	1260	1249
15	21.180	Geranial	9.63	8.99	1275	1264
16	24.010	Acetate of citronella	0.54	0.40	1355	1350
17	25.075	Acetate of geraniol	1.45	1.22	1386	1379
18	26.550	E-Caryophyllene	0.34	0.52	1430	1417
19	31.910	Caryophyllene oxide	0.28	0.41	1597	1582
20	39.635	NI	1.97	2.21	1844	NI

RT = retention time, GC-MS = gas chromatography coupled to mass spectrometry, GC/FID = gas chromatography with flame ionization detector, IRR exp.^*∗*^ = experimental retention index, IRR lit.^*∗∗*^ = retention index found in literature, and NI = not identified.

**Table 2:** Percentages of mass loss and steps of thermal decomposition of the LEO, *β*-cyclodextrin (*β*-CD), physical mixture (PM), and LEO/*β*-CD (CE) complex.

	Mass loss %			
	1st stage	2nd stage	3rd stage	4th stage
LEO	93.94^#^	—	—	—
*β*-CD	12.51^*∗*^	—	72.88^*∗∗*^	10.76^*∗∗∗*^
PM	21.06^+^	1.99^++^	67.22^*∗∗*^	11.20^*∗∗∗*^
CE	13.39^+^	18.02^++^	54.48^*∗∗*^	10.86^*∗∗∗*^

^#^Percentage of leaf essential oil of *C. winterianus* (LEO) evaporated to 291°C. ^*∗*^Percentage of released water to 120°C. ^+^Loss related to the evaporation of the LEO and release of water up to 120°C mass. ^++^Loss attributed to the release of LEO weight in the range 120–270°C. ^*∗∗*^Thermal decomposition in the range 270–365°C. ^*∗∗∗*^Training elemental carbon due to charring in the range 365–900°C.
